# High-Performance Pure Sine Wave Inverter with Robust Intelligent Sliding Mode Maximum Power Point Tracking for Photovoltaic Applications

**DOI:** 10.3390/mi11060585

**Published:** 2020-06-11

**Authors:** En-Chih Chang

**Affiliations:** Department of Electrical Engineering, I-Shou University, No.1, Sec. 1, Syuecheng Rd., Dashu District, Kaohsiung City 84001, Taiwan; enchihchang@isu.edu.tw; Tel.: +886-7-657-7711 (ext. 6642); Fax: +886-7-657-7205

**Keywords:** maximum power point tracking (MPPT), flutter, robust intelligent sliding mode, pure sine wave inverter, enhanced cuckoo optimization algorithm (ECOA)

## Abstract

Photovoltaic (PV) power generation has been extensively used as a result of the limited petrochemical resources and the rise of environmental awareness. Nevertheless, PV arrays have a widespread range of voltage changes in a variety of solar radiation, load, and temperature circumstances, so a maximum power point tracking (MPPT) method must be applied to get maximum power from PV systems. Sliding mode control (SMC) is effectively used in PV power generation due to its robustness, design simplicity, and superior interference suppression. When the PV array is subject to large parameter changes/highly uncertain conditions, the SMC leads to degraded steady-state performance, poor transient tracking speed, and unwanted flutter. Therefore, this paper proposes a robust intelligent sliding mode MPPT-based high-performance pure sine wave inverter for PV applications. The robust SMC is designed through fast sliding regime, which provides fixed time convergence and a non-singularity that allows better response in steady-state and transience. To avoid the flutter caused by system unmodeled dynamics, an enhanced cuckoo optimization algorithm (ECOA) with automatically adjustable step factor and detection probability is used to search control parameters of the robust sliding mode, thus finding global optimal solutions. The coalescence of both robust SMC and ECOA can control the converter to obtain MPPT with faster convergence rate and without untimely trapping at local optimal solutions. Then the pure sine wave inverter with robust intelligent sliding mode MPPT of the PV system delivers a high-quality and stable sinusoidal wave voltage to the load. The efficacy of the proposed method is validated on a MPPT pure sine wave inverter system by using numerical simulations and experiments. The results show that the output of the proposed PV system can improve steady-state performance and transient tracking speed.

## 1. Introduction

The petrochemical energy indispensable to produce electricity has increasingly depleted. The environmental protection awareness has risen, and the application of solar photovoltaic (PV) cells is becoming more and more prevalent. How to improve the conversion efficiency of PV energy and output accessible power will be significant issues for PV power generation applications. For the sake of achieving the maximum efficiency of the PV power generation system, the DC (direct current)–DC (direct current) converter is used to process maximum power point tracking (MPPT) and regulate the voltage of DC load. When the connection of the grid exists, the power is created via solar panel and converted from DC (direct current)–AC (alternating current) pure sine wave inverter to AC power [1,2,3,4]. Therefore, a MPPT-based high-performance pure sine wave inverter must be required to get low AC output-voltage total harmonic distortion (THD), and speedy dynamic behavior. Many MPPT control methods have been put forward to fulfill these requirements, including disturbance observation method, linear iteration method, fuzzy control method, etc. [5,6,7,8]. However, the changes in the maximum power output of PV panels are virtually related to solar illumination and ambient temperature. Most of these MPPT algorithms lack a strict stability and convergence analysis, and only provide close to the maximum power point. Sliding mode control (SMC) has been known to be insensitive to changes in internal parameters and external interferences [9,10,11,12]. That is during its sliding motion, the system trajectory is robust to uncertain intrusions; A great deal of SMC publishing literature has been applied to the control of PV systems [13,14,15,16]. It is worth noting that the PV system is affected by assorted environments (temperature, humidity, illumination, and load) and extremely nonlinearities. At this time, the stability of the convergence and performance of the system will be significantly degenerated. This paper proposes a robust intelligent SMC with a simple architecture and a clear design methodology for MPPT-based high-performance pure sine wave inverters. The robust SMC using nonlinear regime not only ensures that the system state can reach the sliding surface in a limited time, but also demonstrates the capability to suppress severe intrusions in a closed-loop feedback system, which can allow the control more accurate and ensure the stability of the system [17,18,19,20]. The steady-state performance and tracking speed during transients in MPPT inverter system can be improved indeed [21,22,23]. Troublesomely, the high-frequency flutter problem still exists in the robust SMC. This problem may trigger unmodeled plant dynamics, and sometimes even effectuates system instability. The adaptability of the system may depreciate and the adjustment of control parameters is arduous, resulting in unsatisfactory control effect in the PV system. Several methodologies have been proposed to ameliorate flutter problems, such as observer schemes and smart control methods. Notwithstanding that these methodologies dwindle the flutter and mend the transient response under external intrusions and unmodeled dynamics, there are either time-consuming or calls for elaborated mathematics [24,25,26,27]. To slacken the impact of the flutter, an enhanced cuckoo optimization algorithm (ECOA) is used to automatically adjust the control parameters of robust SMC, thus maintaining PV system’s splendid performance. The cuckoo optimization algorithm (COA) is a swarm intelligence technology proffered in 2009. The unsophisticated structure, speedy convergence speed and easy completion disclose its characteristics [28,29,30,31,32,33,34]. However, the traditional COA cannot well adjust the control parameters during the feedback control search process, which gives rise to the high-frequency nonlinear factors excited by the flutter of robust SMC. This results in greater changes in system parameters and affects control performance. The traditional COA degrades the convergence rate and prematurely falls into the local optimal solution. There are some approaches that have been tried to solve this problem, such as Tabu search schemes and Greedy algorithms [35,36,37,38,39]. The Tabu search can find a better solution except slow solution speed and long search time. The solution speed of Greedy algorithm is swift, but it is limited to local search and there is a flaw in easy convergence to local solution. Therefore, an enhanced COA (ECOA) is proposed to meliorate the step size factor and the discovery probability of the traditional COA. That is, this paper uses the ECOA to detect the best global solution in the control parameters of robust SMC, and then furnishes the PV maximum power tracking system with great adaptability. The proposed method is simple to understand, easy to program, fast to converge, and effective in slackening flutter. It leads to a more precise tracking control and a more robust and stable system. Computer simulation and experimentation results uncover that the proposed method enables the PV maximum power tracking system to improve the steady-state efficiency and transient tracking speed, even under non-matching intrusions or in highly uncertain circumstances. The proposed PV maximum power tracking system is also compared with linear sliding regime-based sliding mode controlled PV maximum power tracking system, exposing that the proposed system possesses superior performance.

## 2. Dynamic Modeling of MPPT-Based Pure Sine Wave Inverter

**Figure 1:**
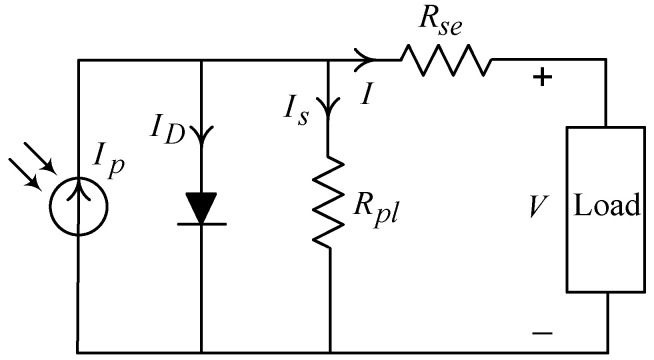
Equivalent circuitry of single solar cell.

Figure 1 discloses the equivalent circuit of a solar cell, which $I_{p}$ is a current source, $I_{D}$ represents a saturation current flowing through a diode, $I_{s}$ stands for a leakage current, $R_{pl}$ denotes an equivalent parallel resistance, $R_{se}$ signifies an equivalent series resistance, I indicates an output current, and V symbolizes an output voltage.

The output current of a single solar cell can be formulated as

(1) $$I=I_{p}-I_{sa}\cdot (e^{K_{o}(V+IR_{se})}-1)-(V+IR_{se}/R_{pl})$$

where $K_{o}=A_{s}/(B_{s}KT)$, $A_{s}$ stands for the amount of charge contained in an electron, $B_{s}$ represents the ideal factor of a solar cell (1 to 5), K denotes the Boltzmann constant, T is the absolute temperature, and the reverse saturation current can be written as $I_{sa}=I_{rs}\cdot {\left(T/T_{ref}\right)}^{3}e^{K_{o}E_{gp}(1/T_{ref}-1/T)}$; here, $I_{rs}$ indicates the reverse saturation current at reference temperature $T_{ref}$ and $E_{gp}$ is the energy gap of the semiconductor material. The current source $I_{p}$ yielded by a solar cell changes with sunlight intensity and ambient temperature variations that is expressed as follows, $I_{p}=[I_{scc}+K_{i}(T-T_{ref})]\cdot L_{s}/1000$; here, $I_{scc}$ implies the short-circuit current of the solar cell under the reference temperature and illumination condition $1000\;{W/m}^{2}$, $K_{i}$ means the temperature coefficient during the short-circuit current of the solar cell (mA/°C), and $L_{s}$ connotes for the sunlight intensity (${\mathrm{kW}/m}^{2}$). 

By specifying the output current $i_{pv}$ and output voltage $v_{pv}$ of the solar array, the PV output power yields

(2) $$P_{pv}=n_{p}i_{p}v_{pv}-n_{p}I_{sa}v_{pv}\cdot (e^{K_{o}v_{pv}/n_{s}}-1)$$

where $n_{p}$ signifies the number of solar cells connected in series and $n_{s}$ points to the number of solar cells connected in parallel. The solar cell output-power can be expressed as

(3) $$P_{pv}=i_{pv}v_{pv}$$

The maximum power point satisfies the following,

(4) $$\partial P_{pv}/\partial v_{pv}=i_{pv}+v_{pv}\cdot \partial i_{pv}/\partial v_{pv}=0$$

**Figure 2:**
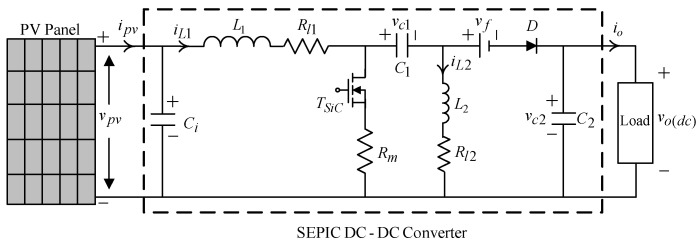
Circuitry structure of SEPIC (single-ended primary-inductor converter) DC-DC converter.

The Equation (4) deduces that after repeated adjustment, a reference voltage value $x_{1}^{ref}$ derived from incremental conductance method is close to a maximum power point voltage $v_{pv}^{\max}$, and then attains to the maximum output. A SEPIC (single-ended primary-inductor converter) DC-DC converter can be utilized to adjust the maximum power point voltage is illustrated as Figure 2, where the capacitor $C_{i}$ aims to enhance response, $R_{l1}$ symbolizes the equivalent internal resistance of the $L_{1}$, $v_{f}$ is the forward conduction voltage of power diode, $R_{m}$ represents the equivalent internal resistance of the power transistor switch, $i_{o}$ stands for output current, and $v_{o(dc)}$ signifies DC output voltage. 

**Figure 3:**
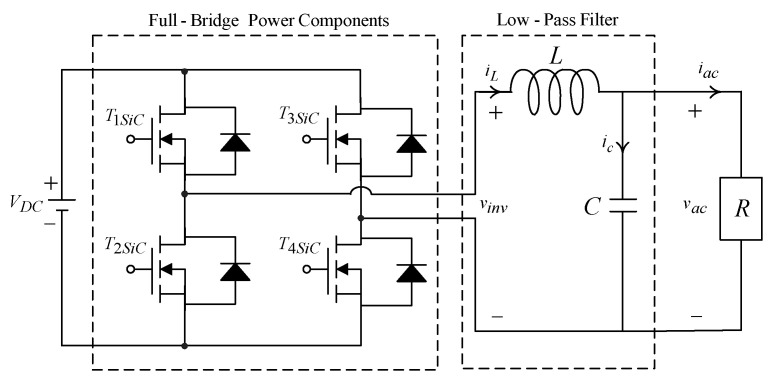
Circuitry construction of single-phase DC–AC inverter.

From the Figure 2, and using the state-space averaging method, one yields

(5) $$\{\begin{array}{l} {\dot{x}}_{1}=\left(i_{pv}-x_{2}\right)/C_{i}\\ {\dot{x}}_{2}=L_{1}^{-1}[(x_{1}-R_{l1}x_{2}-v_{c1}-v_{c2}-v_{d})+(R_{m}x_{2}-R_{m}i_{L2}+v_{c1}+v_{c2}+v_{f})u] \end{array}$$

where $x_{1}=v_{pv}$ and $x_{2}=i_{L1}$ denote state variables, and u is the control signal of the converter. The $x_{1}$ tracking the reference voltage value $x_{1}^{ref}$ is the control target. However, the output response is affected by the external load intrusions, the interventions of sunshine and temperature changes, and the nonlinearities of the converter components. As above-noted uncertainties are considered, a robust SMC with an ECOA for a MPPT-based pure sine wave inverter is designed to produce speedier and more robust output response. Also, Figure 3 depicts a single-phase pure sine wave inverter composed of SiC (silicon carbide) power MOSFET (metal-oxide-semiconductor field-effect transistor), low-pass filter and loading. The L, C, and R symbolize the inductor, capacitor and load, respectively. The $v_{ac}$ stands for the AC output-voltage, $i_{ac}$ infers the output current, and $v_{inv}$ is the pulse-width modulation voltage of magnitude $V_{DC}$ or $-V_{DC}$, with $\Delta T_{sa}$ centered in the sampling interval $T_{sa}$. The $v_{ac}$ must follow a necessitated sinusoidal waveform $v_{ac,\;ref}=V_{m}\sin(\omega t)$, here $V_{m}$ and ω are the peak value and the angular frequency, respectively. By the use of the Kirchhoff’s voltage law and Kirchhoff’s current law, the dynamics of the inverter can be generated as ${\ddot{v}}_{ac}=-v_{ac}/LC-{\dot{v}}_{ac}/RC+v_{inv}/LC$. Then, the tracking error ${\tilde{e}}_{1}=v_{ac}-v_{ac,\;ref}$ and its derivative ${\dot{\tilde{e}}}_{1}={\tilde{e}}_{2}={\dot{v}}_{ac}-{\dot{v}}_{ac,\;ref}$ are obtained. The error dynamic equations of the inverter can be expressed as ${\dot{\tilde{e}}}_{2}=-{\tilde{e}}_{1}/LC-{\tilde{e}}_{2}/RC+u_{in}/LC-v_{ac,ref}/LC-{\dot{v}}_{ac,ref}/RC-{\ddot{v}}_{ac,ref}$; here, $u_{in}=v_{inv}\approx (\Delta T_{s}/T_{s})\cdot V_{DC}$ stands for the control signal of the inverter. To ensure the tracking error converged to zero, the $u_{in}$ is designed by employing proportional–integral scheme formulated as $K_{P}{\tilde{e}}_{1}(kT_{s})+K_{I}\sum_{j=0}^{kT_{s}}{\tilde{e}}_{1}(j)$; here, $T_{s}$ represents sampling period, $K_{P}$ denotes proportional gain, and $K_{I}$ means integral gain. 

## 3. Proposed Controller

It can be seen that the output-voltage of the solar PV cells will be the same as the demanded reference voltage, even if the PV maximum power tracking system occurs under violent external intrusions or great internal parameter changes or rigorous uncertainties. Based on the conception of terminal attractor [17,22,23], this section derives the control law u of the robust SMC, and use the ECOA to unearth the globally optimal parameters of the robust SMC. The MPPT-based pure sine wave inverter can meliorate the performance in steady state and the tracking speed in transient state, thus carrying out the high-quality AC output. From the system dynamics (5) and defining the tracking error $e_{1}$ and its derivative ${\dot{e}}_{1}$, the following error-state equation can be stated as

(6) $$\{\begin{array}{l} {\dot{e}}_{1}=e_{2}={\dot{x}}_{1}-{\dot{x}}_{1}^{ref}\\ {\dot{e}}_{2}=C_{i}^{-1}[({\dot{i}}_{pv}-f(x)-g(x)u]-{\ddot{x}}_{1}^{ref}+d(t) \end{array}$$

where $f(x)=L_{1}^{-1}(x_{1}-R_{l1}x_{2}-v_{c1}-v_{c2}-v_{f})$, $g(x)=L_{1}^{-1}(R_{m}x_{2}-R_{m}i_{L2}+v_{c1}+v_{c2}+v_{f})$, and d(t) signifies the sunshine and temperature variations as well as external load intrusions. The d(t) is restricted to ‖d(t)‖≤w, here w is positive constant.

The sliding regime of a nonsingular robust SMC is constructed as

(7) $$\sigma =e_{1}+(1/\rho )\cdot e_{2}^{z_{2}/z_{1}}$$

where ρ is positive real number, and $z_{1}$ as well as $z_{2}$ advise positive odd numbers which agrees with $1<z_{2}/z_{1}<2$. 

A power reaching law of sliding mode is designed as

(8) $$\dot{\sigma }=-\eta_{1}{\left|\sigma \right|}^{s_{1}}sat(\sigma )-\eta_{2}{\left|\sigma \right|}^{s_{2}}sat(\sigma )-\eta_{3}{\left|\sigma \right|}^{s_{3}}\sigma$$

where $\eta_{1}>0$, $\eta_{2}>0$, $\eta_{3}>0$, $0<s_{1}<1$, $s_{2}>1$, $s_{3}>0$, and a saturation function sat(σ)=σ/Δ, 1, −1 for −Δ<σ≤Δ, σ≥Δ, σ<−Δ (0<Δ<<1), respectively.

From the Equations (6)–(8), the control law of robust SMC becomes

(9) $$u(t)=-g{\left(x\right)}^{-1}[f(x)-{\dot{i}}_{pv}+C_{i}{\ddot{x}}_{1}^{ref}+(z_{1}/\rho z_{2})\cdot e_{2}^{2-z_{2}/z_{1}}-\eta_{1}{\left|\sigma \right|}^{s_{1}}sat(\sigma )-\eta_{2}{\left|\sigma \right|}^{s_{2}}sat(\sigma )-\eta_{3}{\left|\sigma \right|}^{s_{3}}\sigma ]$$

**Theorem** **1.** *For the system dynamics (6), once the control is adopted as the Equation (9) with the sliding regime σ in the Equation (7) and a sliding-mode power reaching law (8), the swift nonsingular convergence of the system state to the equilibrium in finite time will be fulfilled.*

**Proof.**  Choose the following Lyapunov function

(10) $$V=\sigma^{2}/2$$

Get the time derivative for Equation (10):

(11) $$\begin{array}{cc} \dot{V} & =\sigma \dot{\sigma }\\ & =\sigma \left({\dot{e}}_{1}+(z_{2}/\rho z_{1})\cdot e_{2}^{z_{2}/z_{1}-1}{\dot{e}}_{2}\right)\\ & \leq -(z_{2}/\rho z_{1})\cdot e_{2}^{z_{2}/z_{1}-1}[(\eta_{1}/\Delta ){\left|\sigma \right|}^{s_{1}+2}+(\eta_{2}/\Delta ){\left|\sigma \right|}^{s_{2}+2}+\eta_{3}{\left|\sigma \right|}^{s_{3}+2}-w\left|\sigma \right|)] \end{array}$$

Because σ and $e_{2}$ do not equal zero, $\dot{V}$ is smaller than zero. Equation (11) conforms to Lyapunov’s stability theorem, and the robust SMC system briskly converges to the equilibrium region within the limited time. It can be found more subtly that the $-\eta_{1}{\left|\sigma \right|}^{s_{1}}sat(\sigma )$ and $-\eta_{2}{\left|\sigma \right|}^{s_{2}}sat(\sigma )$ signify the system dynamic behavior near the sliding regime and away from the sliding regime, respectively; the $\eta_{1}$ and $\eta_{2}$ gains infer that the nonlinearities formulated in Equation (5) can be compensated, showing the characteristic of robust SMC against system uncertainties. Once the varied illumination, loading, temperature and humidity, and uncertain nonlinearity impact on the PV system, the high-frequency flutter or steady-state error occurs and may incur tracking imprecision. The adjustment of the robust SMC parameters presents the obstacle and the system adaptability declines. By the use of the ECOA, flutter relief and swiftness search are accomplished. The control parameters of the robust SMC can be adaptively adjusted to acquire the best solution, avoiding premature falling into the local’s best solution.  □

The COA uses the following two mechanisms to generate offspring,

(12) $$\{\begin{array}{l} x^{k+1}=x^{k}+\beta \cdot \zeta_{l}⊗(x_{i}^{k}-x_{h})⊗r_{h}\\ x_{i}^{k+1}=x_{i}^{k}+r_{g}\cdot (x_{m}^{k}-x_{z}^{k})⊗H_{e}(p_{a}-r_{g}) \end{array}$$

where β is the step size factor; $\zeta_{l}$ stands for the L´evy distribution [40,41,42]; $x_{h}$ signifies the historical best solution; $r_{h}$ indicates the normal distribution; $x_{i}$, $x_{m}$, and $x_{z}$ denote random select solutions; $r_{g}$ infers the uniform distribution on [0, 1], $H_{e}(\cdot )$ symbolizes the Heaviside function [43,44,45]; and $p_{a}$ means the discovery probability. The next-generation solution of the COA can reflect the configuration of the population; it is passive and does not the capability to dynamically learn and adapt during the searching process. The flutter phenomenon agitates the high-frequency nonlinear factors, which yield greater parametric changes. The control performance and accuracy are swayed, thereby displaying slow convergence rate in unearthing solution and falling into the local optimum. The step size factor and discovery probability can be adjusted to effectuate speedy convergence speed, and uncover the solution of global optimization. The step size factor $\beta^{k+1}$ and discovery probability $p_{\beta }^{k+1}$ changing with step size factor of the ECOA are restructured as

(13) $$\beta^{k+1};\;p_{\beta }^{k+1}=\{\begin{array}{l} \beta^{k}L_{\beta };\;p_{\beta }^{k}L_{p},P_{r}>\Delta \\ \beta^{k};\;p_{\beta }^{k},\Delta \leq P_{r}\leq 2\Delta \\ \beta^{k}L_{\beta }^{-1};\;p_{\beta }^{k}L_{p}^{-1},P_{r}<\Delta \end{array}$$

where $L_{\beta }$ is the step size learning factor, and $L_{p}$ indicates the discovery probability learning factor, $P_{r}$ denotes the proportion of new solution, and Δ=0.01 represents the boundary layer thickness of the saturation function. The step flow of the ECOA is depicted as follows. Step 1: Initialize the population and count the fitness of all individuals. Step 2: For each individual, engender a new solution according to the Equation (12). If the new solution is preferable to the old solution, supersede the old solution and augment the number of the improvement. Step 3: For each individual, a new solution is engendered in accordance with the similarity and discovery probability. If the new solution is better, the old solution is supplanted; the number of the improvement increases while the already improved solution is no longer calculated repeatedly. Step 4: Count the proportion of the improved individuals in the population, and decide the step size factor and discovery probability of the next-generation population according to the Equation (13). Step 5: Enroll the best solution. If the termination condition is not met, reiterate Step 2 to Step 4. From the mathematical derivation and proof of robust SMC described in Equations (9) and (11), they represent the equivalent control term with non-singularity, and the sliding control term with intrusion compensation. Equations (7) and (8) can be allowed to converge to the equilibrium for a limited time. Then, the system dynamics (5) will also be converged to the equilibrium for a limited time while the sliding regime is approached. Eventually, the robust SMC parameters can be adaptively adjusted through the ECOA expressed in the Equation (13), so as to ensure the stability of the global system subject to parameter changes and uncertain intrusions.

## 4. Results and Analysis

**Figure 4:**
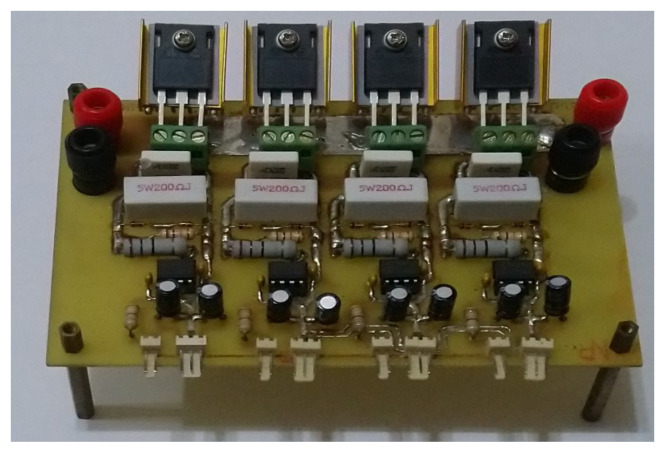
Hardware photography of sine-wave conversion circuitry.

The proposed method and MPPT-based high-performance pure sine wave inverter are modeled and simulated by SimPowerSystems (version 3.1), which utilizes the Simulink environment. In addition, the circuitry structure has been framed and tested, implementing the proposed control technology with a TI-DSP (Texas Instruments-Digital Signal Processor, Dallas, TX, USA). The system parameters of the SEPIC DC-DC converter and single-phase DC-AC Inverter are shown in Table 1, and the conversion circuitry of the main sine wave is revealed as the hardware experimental set-up of the Figure 4.

**Figure 5:**
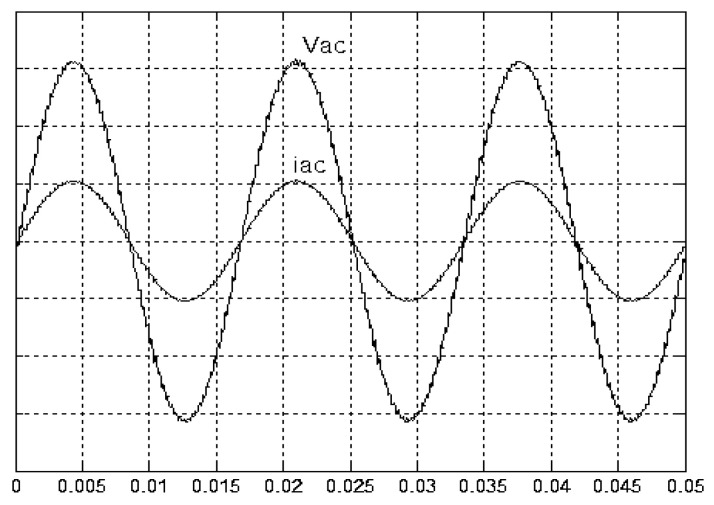
Simulation output voltage and current obtained using the linear sliding regime-based sliding mode control (SMC) under full resistive load (vertical: 50 V/division and 15 A/division).

**Figure 6:**
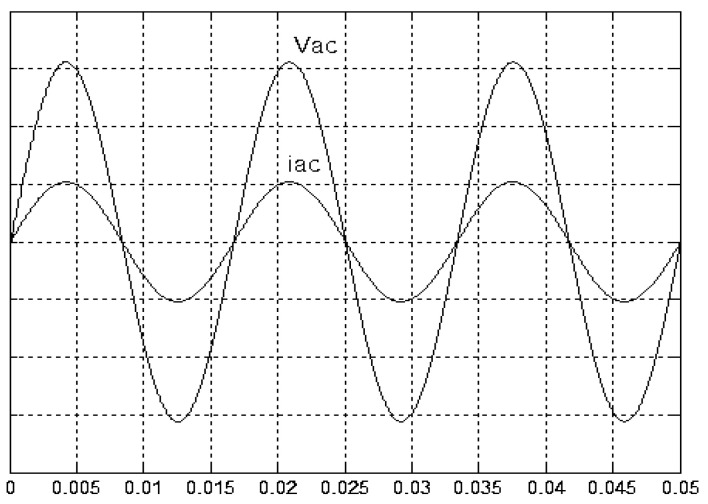
Simulation output voltage and current obtained using the proposed method under full resistive load (vertical: 50 V/division and 15 A/division).

**Figure 7:**
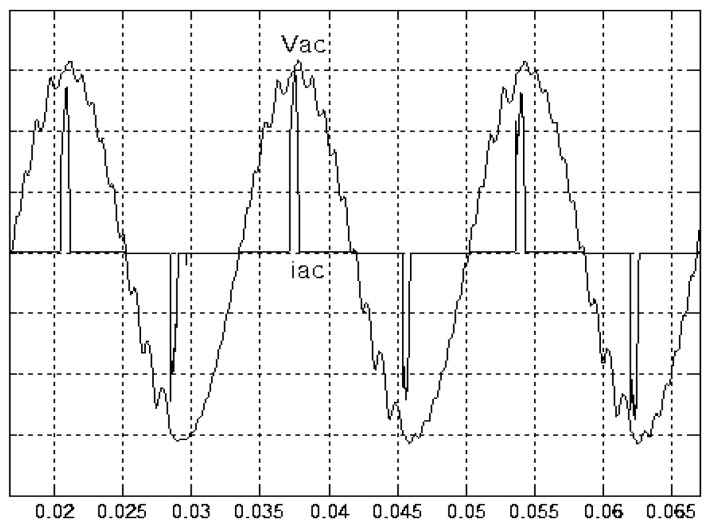
Simulation output voltage and current obtained using the linear sliding regime-based SMC under rectifier load (vertical: 50 V/division and 25 A/division).

**Figure 8:**
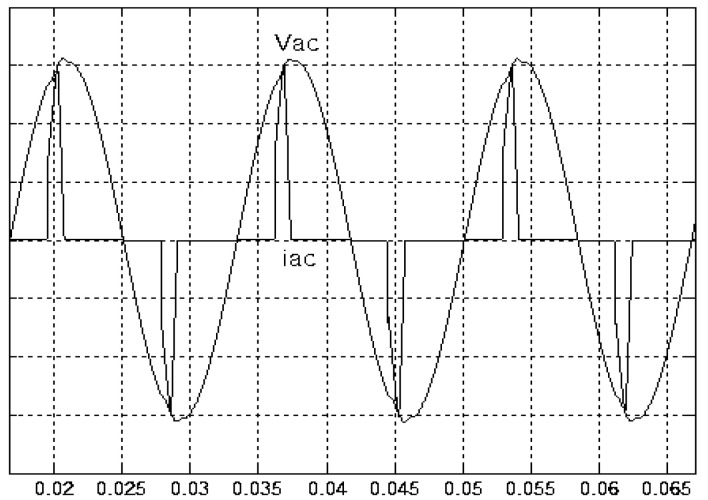
Simulation output voltage and current obtained using the proposed method under rectifier load (vertical: 50 V/division and 25 A/division).

**Figure 9:**
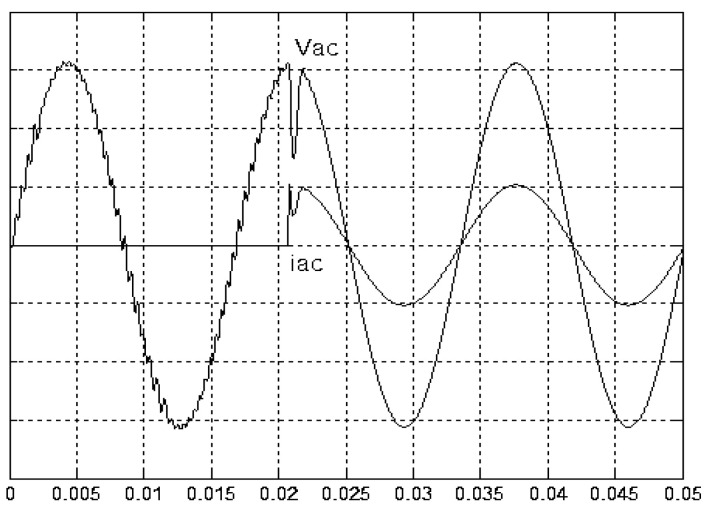
Simulation output voltage and current obtained using the linear sliding regime-based SMC under step load change (vertical: 50 V/division and 15 A/division).

**Figure 10:**
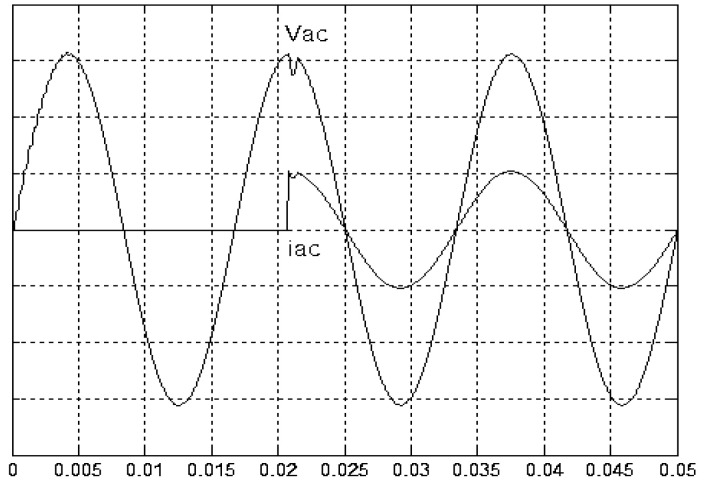
Simulation output voltage and current obtained using the proposed method under step load change (vertical: 50 V/division and 15 A/division).

**Figure 11:**
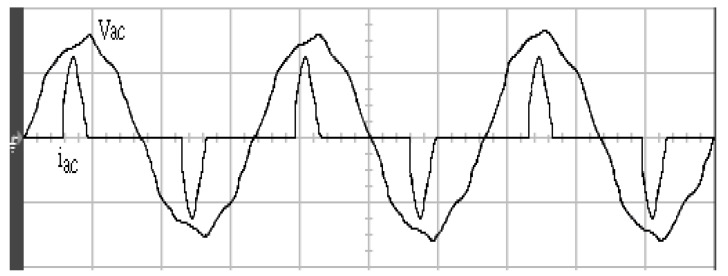
Experimentation output voltage and current obtained using the linear sliding regime-based SMC under rectifier load (vertical: 100 V/division and 10 A/division; horizontal: 5 ms/division).

**Figure 12:**
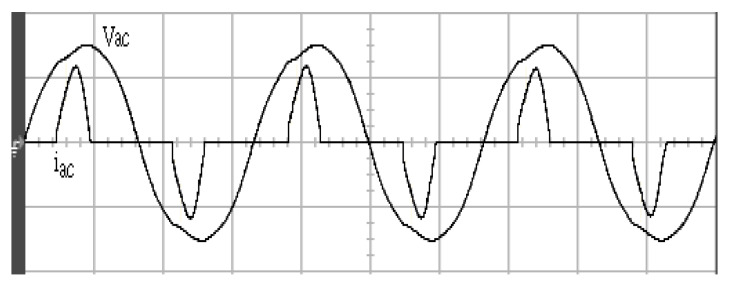
Experimentation output voltage and current obtained using the proposed method under rectifier load (vertical: 100 V/division and 10 A/division; horizontal: 5 ms/division).

**Figure 13:**
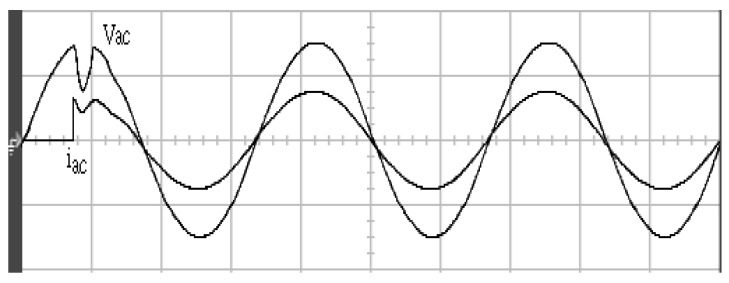
Experimentation output voltage and current obtained using the linear sliding regime-based SMC under step load change (vertical: 100 V/division and 20 A/division; horizontal: 5 ms/division).

**Figure 14:**
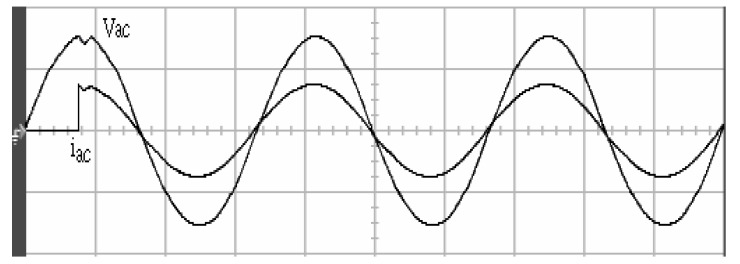
Experimentation output voltage and current obtained using the proposed method under step load change (vertical: 100 V/division and 20 A/division; horizontal: 5 ms/division).

**Figure 15:**
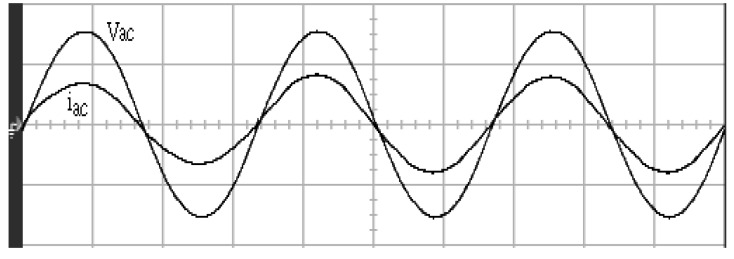
Experimentation output voltage and current obtained using the proposed method form 12-ohm to 10-ohm at 1/60 s (vertical: 100 V/division and 20 A/division; horizontal: 5 ms/division).

**Figure 16:**
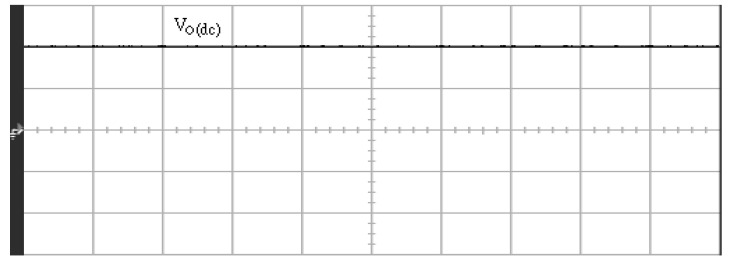
Experimentation DC voltage in the DC side of the inverter (vertical: 100 V/division; horizontal: 5 ms/division).

**Figure 17:**
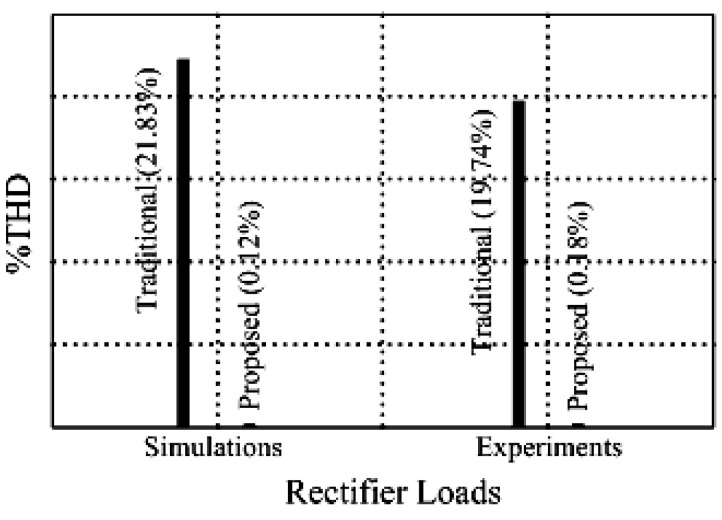
Comparison of harmonic distortion.

**Figure 18:**
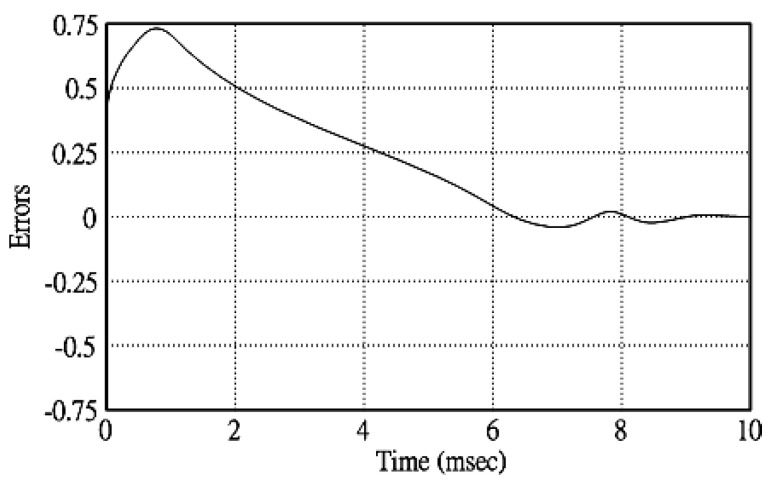
Tracking error obtained using the linear sliding regime-based SMC.

**Figure 19:**
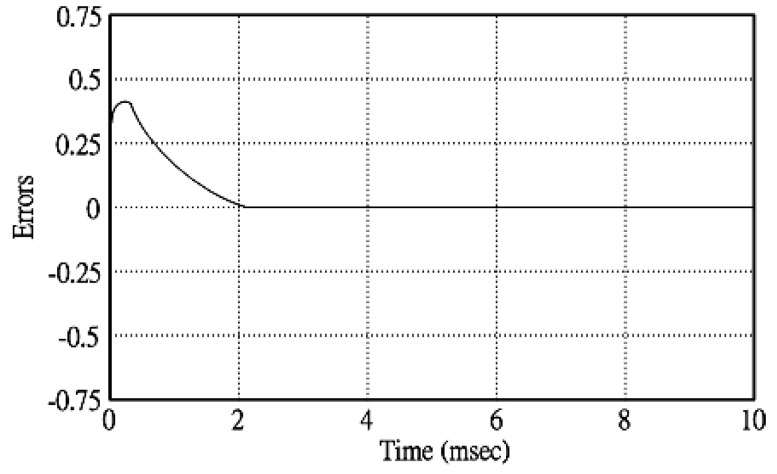
Tracking error obtained using the proposed method.

Figure 5 and Figure 6 exhibit the simulation output-waveforms of the linear sliding regime-based sliding mode controlled PV system (%THD (total harmonic distortion)) = 1.6%) and the proposed PV system (%THD = 0.05%) under full resistive loads. Though both output voltage are sine form, the linear sliding regime-based SMC PV system causes distorted voltage while the proposed PV system uncovers undistorted steady-state response. Figure 7 is the simulation output-waveforms of the PV system obtained using the linear sliding regime-based SMC under the rectifier load circumstance. It can be seen from the figure that the output-voltage is an excessively contorted sinusoidal waveform, and the reckoned %THD has a high %THD value of 21.83%. Figure 8 depicts the simulation output-waveforms of the PV system obtained using the proposed method under the rectifier load. A great spike current is unearthed, but the output-voltage waveform is just about the necessitated sinusoidal reference voltage (low %THD value of 0.12%). It can be perceived that under the case of rectified load, the proposed PV system brings preferable steady state respondence than the linear sliding regime-based SMC PV system. Figure 9 expounds the simulation output-waveforms of the PV system obtained using the linear sliding regime-based SMC under step load changes from no load to full load at a trigger angle of 90 degree. The large momentary voltage drop happens, i.e., the compensation capability of the linear sliding regime-based SMC remains to be reinforced. Figure 10 manifests the simulation output-waveforms of the PV system obtained using the proposed method under the same loading environment at a trigger angle of 90 degree. The little voltage drop is remarked and the voltage recuperates to the desired sine level for a very short time, pointing to pleasing transient respondence. Figure 11 depicts the experimentation output-waveform of the PV system controlled by the linear sliding regime-based SMC under the condition of rectifier load. The output-voltage shows a contortion of the sine wave, and its measured %THD takes on a high value of 19.74%. Figure 12 recites the proposed experimentation output-waveforms at rectifier type load. A pure sine-wave steady-state response (0.18% voltage THD) with the necessitated sinusoidal form voltage is seen, even though a sharply rising current occurs. The PV system controlled by the linear sliding regime-based SMC at a 90-degree trigger angle, changing from no load to full load yields the experimentation output-waveforms of the Figure 13. Owing to the tardy retrieve of the sudden voltage drop, the transient output-voltage discloses defective behavior response. Figure 14 illustrates the experimentation output-waveforms of the proposed PV system, when it confronts load-changing from no load to full load at 90-degree trigger angle; we can make out that the strong sine trajectory is non-oscillatory before and after speedy recovery voltage drop. To verify once again the dynamic performance of the proposed method, Figure 15 displays the experimentation output waveforms from 12-ohm to 10-ohm conditions at 1/60 s, confirming exceptional transience response. In the DC side of the inverter, the experimentation output-voltage of the DC–DC converter is shown in Figure 16. With the use of the Agilent E4360A solar simulator, the PV modules can be emulated and then demonstrated the system operation. It can be clearly observed from the output-voltage that the nearly ripple-free DC waveform has been obtained. This verifies that even if the PV module is disturbed, the DC-DC converter can still provide a stable and robust DC output to the DC-AC inverter. Figure 17 plots a comparison of the %THD obtained using the linear sliding regime-based SMC and proposed method under rectifier loads. Figure 18 and Figure 19 are the tracking errors of the linear sliding regime-based SMC and proposed method. Distinctly, as compared with the linear sliding regime-based SMC, the proposed method holds high-precision tracking and limited-time convergence characteristics. In other words, in the early stage of the proposed algorithm, the learning value of the step size is large enough and the value of the discovery probability is relatively small to enhance the diversity of the solution. On account of the update of the relatively large number in the bird nests, the proposed algorithm maintains a strong global search capability; in the later stage of the proposed algorithm, the step size learning value cuts down and a relatively large discovery probability value is used to adjust the solution variables and uphold a strong local search capability. In the conclusive synopsis, a comparative analysis with other literature is discussed below. An H-infinity control technology is recommended to ensure the global stability in small signal model for power converters. Its robustness and ascendant performance in steady state as well as transient response can be acquired. Regrettably, this technology has the requirement of intricate calculations [46]. For the sake of overcoming system uncertainties, a higher-order repetitive approach is developed to regulate a two-level grid-connected inverter. This design structure encompassing the phase lead compensator shows the simplification and provides the tracking response of zero-phase error, but the tracking behavior in the transience is not satisfactory [47]. An unsophisticated deadbeat control with the use of a current observer has been presented for hybrid energy storage systems. Although the exceptional performance in dynamics as well as steady state can be obtained, this strategy relies heavily on the preciseness of the system parameters [48]. The mu-synthesis technique attempts to improve the system stability in the wind farm model; this methodology addresses the nonlinear intrusion faced by the system, but it is time-consuming to calculate and has a complex structure is not easy to understand [49].

## 5. Conclusions

The proposed method uses an ECOA to unearth the optimal solution of the robust SMC parameter, effecting its great adaptability to avoid untimely falling into local optimal solution and ameliorate slower convergence speed. The proposed method not only eschews flutter/steady-state error occurred in the robust SMC, but also holds nonsingular limited-time convergence of robust SMC. The robust intelligent sliding mode MPPT-based high-performance pure sine wave inverter can conspicuously provide smaller losses for greater efficiency, higher switching frequency, therefore establishing respectable performance in steady state and tracking speed in transient state under large parameter changes/high uncertainty circumstances. We can compare the results between the linear sliding regime-based SMC and the proposed method, in terms of total harmonic distortion in the face of resistive and rectified loads. Under the situation of resistive load, both linear sliding regime-based robust sliding mode controlled PV system and proposed PV system display low output-voltage %THD, and the voltage-waveforms are approximately the sinusoidal reference voltage. Under the environment of rectified load, the output-voltage of the PV system with linear sliding regime-based SMC produces a very high %THD, but the proposed PV system acquires quite well steady-state response with output-voltage %THD of much smaller than 5%, which excels IEEE standard 519.

## Figures and Tables

**Table 1:** Photovoltaic (PV) system parameters.

**SEPIC DC-DC Converter**	
Inductance	50 μH
Internal resistance	136 mΩ
Input capacitance	330 μF
Output capacitance	1000 μF
**Single-phase DC-AC Inverter**	
Filter inductor	0.2 mH
Filter capacitor	5 μF
Resistive load	12 Ω
DC-link voltage	200 V
AC output voltage	110 V_rms_
AC output-voltage frequency	60 Hz
Switching frequency	24 kHz
